# Photocurrent generation in lateral graphene p-n junction created by electron-beam irradiation

**DOI:** 10.1038/srep12014

**Published:** 2015-07-08

**Authors:** Xuechao Yu, Youde Shen, Tao Liu, Tao (Tom) Wu, Qi Jie Wang

**Affiliations:** 1 OPTIMUS, Photonics Centre of Excellence, School of Electrical and Electronic Engineering, Nanyang Technological University, 50 Nanyang Ave., 639798, Singapore; 2 Centre for Disruptive Photonic Technologies, Nanyang Technological University, 21 Nanyang Link, 637371, Singapore; 3 Materials Sciences and Engineering Division, King Abdullah University of Science and Technology (KAUST), Thuwal, 23955-6900, Saudi Arabia

## Abstract

Graphene has been considered as an attractive material for optoelectronic applications such as photodetectors owing to its extraordinary properties, e.g. broadband absorption and ultrahigh mobility. However, challenges still remain in fundamental and practical aspects of the conventional graphene photodetectors which normally rely on the photoconductive mode of operation which has the drawback of e.g. high dark current. Here, we demonstrated the photovoltaic mode operation in graphene p-n junctions fabricated by a simple but effective electron irradiation method that induces n-type doping in intrinsic p-type graphene. The physical mechanism of the junction formation is owing to the substrate gating effect caused by electron irradiation. Photoresponse was obtained for this type of photodetector because the photoexcited electron-hole pairs can be separated in the graphene p-n junction by the built-in potential. The fabricated graphene p-n junction photodetectors exhibit a high detectivity up to ~3 × 10^10^ Jones (cm Hz^1/2^ W^−1^) at room temperature, which is on a par with that of the traditional III–V photodetectors. The demonstrated novel and simple scheme for obtaining graphene p-n junctions can be used for other optoelectronic devices such as solar cells and be applied to other two dimensional materials based devices.

Graphene, a single layer of carbon atoms arranged in a honeycomb lattice with broadband light absorption spectrum^1^ and ultrahigh carrier mobility^2^, has seen a significant rise in various electronic and optoelectronic applications such as transistors^3^, modulators^4^, photodetectors^5^,^6^. Conventional graphene based photodetectors that rely on the Scottky junctions at the graphene/metal contacts interface, where a built-in potential drives the separation and transport of photogenerated electron-hole pairs, are promising for high speed, broadband and flexible optoelectronic and photonic devices^7^,^8^. However, the symmetric metal-graphene-metal structure in common graphene field effects (FET) with two equal electrodes is not effective to produce photocurrent under global illumination because the metal-graphene interfaces of both sides of the device generate an equal positive and negative current flow in the vicinity of the Schottky junctions^9^. In this context, graphene photodetector with photovoltaic mode operation is of particular importance in practical applications because it yields a low dark current and higher detectivity compared to the conventional photoconductive mode operation and thermoelectric mode operation, without requiring external bias for operations.

Pioneering works have already been reported on building asymmetric potential in graphene sheet, attempting to obtain graphene p-n junctions where the photoexcited electron-hole pairs can be intrinsically separated. For instance, Mueller *et al.*^10^ proposed to utilize distinct electrodes with different work functions to break the symmetric band diagram of metal-graphene-metal structure, but the barrier height caused by metals is theoretically limited and the fabrication process is quite complicated. Dual-gates^11^, substitutional doping^12^,^13^,^14^, chemical dopants^15^,^16^ and plasmon-induced doping^17^,^18^ were also employed to obtain p-n junctions in graphene devices, which however imposes limitations on the device architectures and functionalities. Recently, graphene p-n junctions with an intrinsic potential offset obtained by modulation-doped growth of large-area graphene through the chemical vapor deposition (CVD) process were reported and considerable photocurrents originated from thermoelectric effect were measured in these devices under global illuminations^19^. Furthermore, recent reports demonstrated that it is possible to control the chemical potential and doping level by electron beam or focused ion beam irradiation^20^,^21^.

In this study, we partially irradiated the graphene sheet to obtain an n-type graphene region which is originally p-doped, thus forming a lateral graphene p-n junction. The physical mechanism behind it is due to the interaction of the electron beam with the Si/SiO_2_ interface in the substrate which induces a gating effect on the irradiated graphene. Using this novel but simple strategy, we obtained graphene photodetectors in a field effect transistor (FET) structure with high detectivity and fast response time which were found to be strongly dependent on the electron irradiation. The methodology demonstrated here enables us to modify the transport properties of graphene by electron irradiation, thus paving the way to fabricating graphene homo-junctions controllably and benefiting the future electronic and photonic applications of graphene and other two dimensional (2D) materials based electronic and optoelectronic devices.

## Results and Discussions

Single layer graphene samples were fabricated by mechanical exfoliation of highly ordered pyrolytic graphite (HOPG) on SiO_2_/Si wafer and then verified by Raman spectroscopy (see Methods). We next fabricated single layer graphene FETs with the heavily doped silicon substrate as a backgate electrode by standard photolithography and e-beam evaporation. After that, the FETs were placed in a scanning electron microscope (SEM) under high vacuum and selected area was exposed to the electron beam with controlled low-energy electron beam irradiation (see Methods). The electron beam irradiates part of the graphene sheet and modifies the properties of graphene and the SiO_2_/Si substrate^20^, which is the main mechanism behind the tuning of the electronic properties of graphene FET and will be discussed in details later.

**Figure 1:**
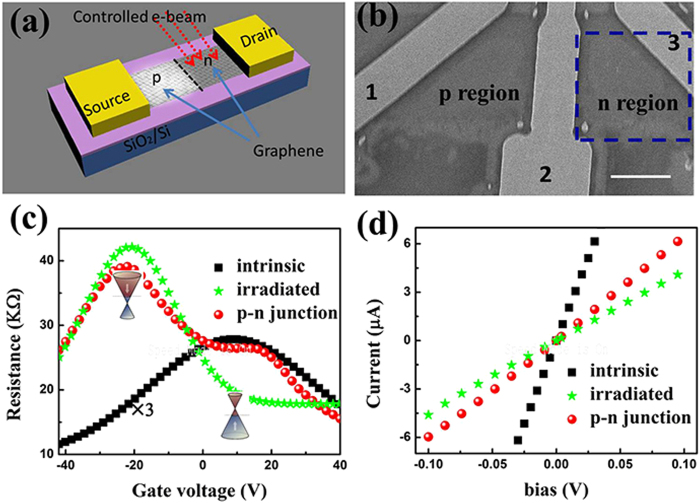
Device fabrication route and electrical characterizations. (**a**) Schematic design of the electron irradiation modulated graphene field effect transistor (FET); (**b**) Scanning electron microscope (SEM) image of the electron irradiation graphene FET, and the squared area are marked for electron irradiation. The scale bar is 10 μm ; (**c**) The electric characteristics as a function of gate bias of the intrinsic graphene FET, the irradiated graphene FET and the fabricated graphene p-n junction; (**d**) Current-voltage (I-V) curves of the same sample in (**a**) at room temperature with zero gate bias. The black, blue and red color lines represent measurements between electrodes 1-2, electrodes 2- 3 and electrodes 1-3 as shown in Fig. 1(b), respectively.

Electrical transport measurements were employed to investigate the effect of electron beam irradiation on the electronic properties of graphene FETs. As shown in Fig. 1(c,d), the pristine graphene obtained in our experiment is p-doped because of the hydrocarbon molecules or humidity absorbed on the graphene surface. In contrary, the Dirac point of electron-irradiated graphene FET shifts to the gate voltage of −20 V, as shown in Fig. 1(c), exhibiting the n-type doping of graphene. It is very obvious that, consistent with previous reports^3^, the on/off ratio of the graphene FET is far below conventional silicon based FET. The carrier mobility of the graphene FETs can be estimated based on the equation^3^: μ = dI_ds_/dV_b_ × L/(W × (ε_0_ε_r_/d) × V_ds_), where L, W and d are the channel length, width and the thickness of SiO_2_ layer (285 nm in our devices), V_ds_, I_ds_ and V_b_ are source-drain bias, current and bottom gate voltage, ε_0_ and ε_r_ are the vacuum dielectric constant and the dielectric constant of SiO_2_ (ε_r_ = 3.9), respectively. The mobility of the n-type region (~1500 cm^2^ V^−1^s^−1^) is several times lower than that of pristine graphene (~5500 cm^2^ V^−1^s^−1^), which might be caused by the change of carrier density and the shift of Fermi level in the graphene channel after the electron irradiation^20^. However, the mobility is higher than that of graphene nanostructures^22^,^23^,^24^ since less edge/boundary scatterings are introduced by the electron beam irradiation method.

**Figure 2:**
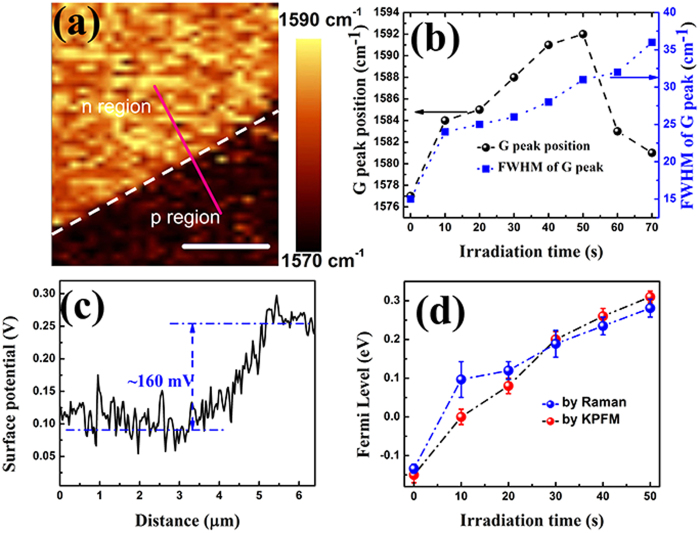
Characterizations of the shift of Fermi level after electron beam irradiation. (**a**) G band mapping of the boundary of irradiated (with 30 seconds treatment) and un-irradiated graphene sample, and the white dash line is the boundary between the irradiated and the un-irradiated regions. The scar bar is 3 μm; (**b**) evolution of the G Raman band position and FWHM with different irradiation times; (**c**) Kelvin Probe Force Microscope (KPFM) profile of the graphene sample along the pink line in Fig. 2(a); (**d**) Fermi level evolution detected by KPFM and calculation from the G peak position.

Fermi level of graphene can be characterized and qualified by the G peak position of graphene. As shown in Fig. 2(a,b), the G peak indicates an obvious blue shift with the irradiation time increasing from 10 s to 50 s and then a red shift with a longer irradiation time. In the meantime, the full width at half maximum (FWHM) of the G peak monotonously increases with the irradiation time. The blue shift of the G peak indicates the n-type doping of graphene and the broadening of FWHM of G peak are also caused by the doping effect. Furthermore, the position evolution of 2D peak indicates the n-doping effect caused by the electron beam irradiation, which is consistent with previous reports^25^. With a longer irradiation time, the doping effect is saturated, consistent with the saturation of the G peak shifting, and then the disorder-induced broadening of the G peak becomes apparent.

The doping effect of electron irradiation on graphene as demonstrated by the G peak shift as shown in Fig. 2(a,b) can be quantized by the following equation^25^:


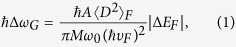


where <*D*^*2*^> is the deformation potential of the E_2g_ mode, M is the atomic weight of carbon, ω_0_ is the frequency of the G-band in perfect graphene, *υ*_*F*_ is the Fermi velocity of graphene, and Δ*E*_*F*_ is the shift of the Fermi level. The blue shift of *G* peak indicates n-type doping resulting from the charge transfer from the treatment or the substrate to graphene^25^. Alternatively, Kelvin probe force microscope (KPFM) is also widely used to characterize the spatial charges distribution of graphene under different doping conditions^26^. A typical potential profile acquired on the sample after 30 s irradiation is shown in Fig. 2(c), and the same sample was used for the electric transport measurement in Fig. 1. The shift of the Fermi level of graphene is around 160 meV, which is consistent with the estimation from equation (1) by the blue shift of *G* peak. Similarly we can plot the Fermi level shifts of the samples with different irradiated times from 10 s to 50 s in Fig. 2(d). We did not conduct experiments with longer irradiation times to avoid the formation of the amorphous graphene.

**Figure 3:**
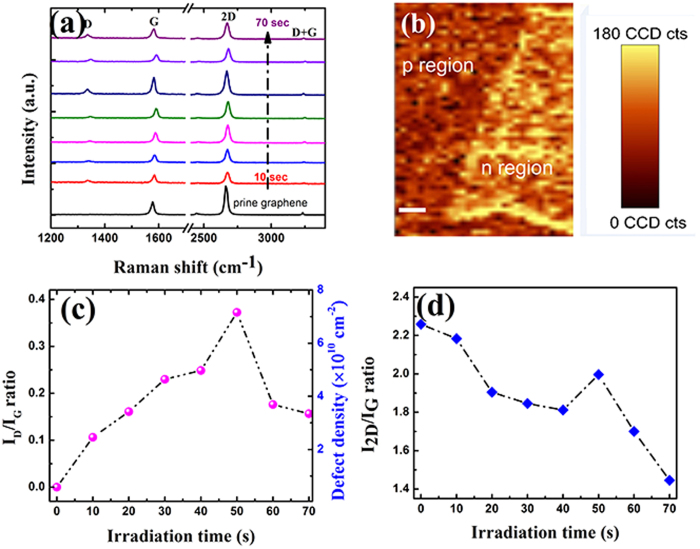
Characterization of defects and defect density by Raman spectroscopy. (**a**) Raman spectra of graphene with different irradiation times from 10 seconds to 70 seconds; (**b**) D band mapping of the boundary of irradiated (with 30 seconds treatment) and un-irradiated regions in the graphene sample; (**c**) Intensity ratios of *I*_*D*_*/I*_*G*_ and defect densities after different irradiation times; (**d**) Intensity ratios of *I*_*2D*_*/I*_*G*_ after different irradiation times.

There are two mechanisms that may contribute to the shift of the Fermi level of graphene from negative (p-doped) to positive (n-doped): defect effect^27^ and substrate gating effect^20^. First, we employed Raman spectroscopy to characterize the quality and the defect effects on graphene properties. Figure 3(a) shows the evolution of Raman spectra of single layer graphene under different irradiation dosage. An important feature is the presence of disorder-induced *D*, *D’* and *D* + *G* bands at ~1350 cm^−1^, 1630 cm^−1^ and 2930 cm^−1^, and their intensities increase with irradiation dosage that controlled by the irradiation time. On the other hand, these three bands are absent in the pristine graphene sample as shown in Fig. 3(a), indicating that the defects or localized disorder were introduced by the electron irradiation. Furthermore, the density of disorder and defects increase with the irradiation dosage. Another feature of the spectra is that the *2D* band at ~2700 cm^−1^ remains symmetric, although the intensity decreases slowly with the irradiation dosage, which indicates the decrease of the quality of graphene under the electron irradiation. The decrease of I_2D_/I_G_ with the irradiation time longer than 50s indicates the amorphization of graphene from sp^2^ to sp^3^ carbon^28^. Hereafter, we will not consider the amorphous graphene devices that are irradiated longer than 50s in our experiments.

However, we note that a relatively large Fermi level shift of ~0.3 eV for the sample after 50 second electron irradiation cannot be totally contributed to the generation of defects in which the excess electrons are localized according to density function theory (DFT) predication and experimental demonstration^29^,^30^. Another important observation is that all the devices after 10s to 50s irradiation times retain a remarkably high mobility, indicating that the honeycomb structure of graphene is preserved and the defect density is quite low. The defect density (n_D_) induced by electron irradiation can be calculated from^29^: 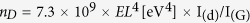, where E_L_ (2.33 eV) is the laser excitation energy, I(D) and I(G) are the intensities of the D band and G band as shown in Fig. 3 (a). The estimated defects densities as shown in Fig. 3(c) are quite low compared to the total carrier density of graphene at zero gate voltage (around 10^13^ cm^−2^). This low defect effect here cannot cause the opening of bandgap of graphene, which is consistent with the density function theory (DFT) calculations in previous works^30^. Thus we can contribute most of the negative shift in the charge-neutral point to the substrate gating effect caused by the electron irradiation as discussed below.

**Figure 4:**
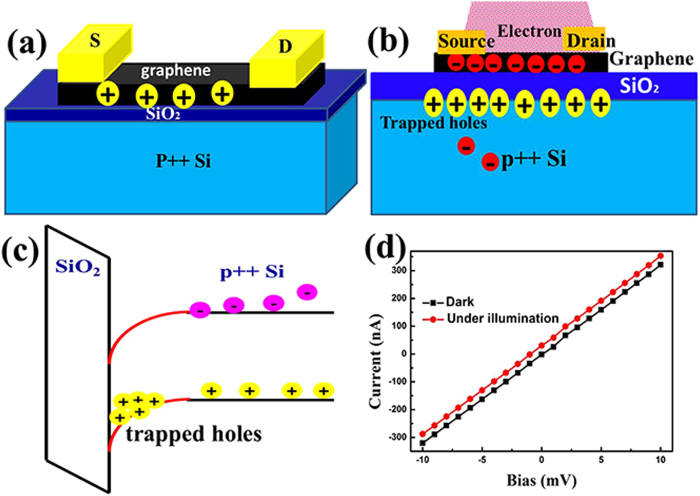
Substrate effect of electron irradiation. (**a**) Schematic structure of the intrinsic p-type graphene FET device; (**b**) Substrate gating effect of the electron irradiation and charge distribution in the graphene FET on Si/SiO_2_ substrate; (**c**) Band diagram of Si/SiO_2_ interface, holes are trapped in the interface; (**d**) I_d_-V_d_ curve in the dark (black square) and under 633 nm laser illumination (red dot).

The main physical mechanism changing the p-doped graphene to n-doped under electron beam irradiation is shown in Fig. 4(b), the high energy electron irradiation introduces electron-hole pairs and the holes are trapped at the SiO_2_/Si interface because of the mismatch of the workfunction of silicon and p-doped silica where the conduction and valence bands in Silicon bend downward at the interface^31^. As a result, the electron irradiation caused the low mobility holes move and get trapped in the triangular potential formed at the Si/SiO_2_ interface^31^ (Fig. 4(c)), leading to an extra positive bias for the bottom gate, which is similar to the previous report on photoinduced voltage at the SiO_2_/Si interface^32^. Therefore, by partially irradiating the graphene FET channel with electron beam, we can reliably obtain a graphene p-n junction as shown in Fig. 1(a). The current versus source-drain bias without illumination and with 633 nm laser illuminations are shown in Fig. 4(d), where an obvious upshift of the I-V curve is observed as the photoexcited electron-hole pairs are separated by the p-n junction and thus photocurrent is generated. Detailed studies of the optoelectronic properties of the obtained graphene p-n junction will be discussed below.

**Figure 5:**
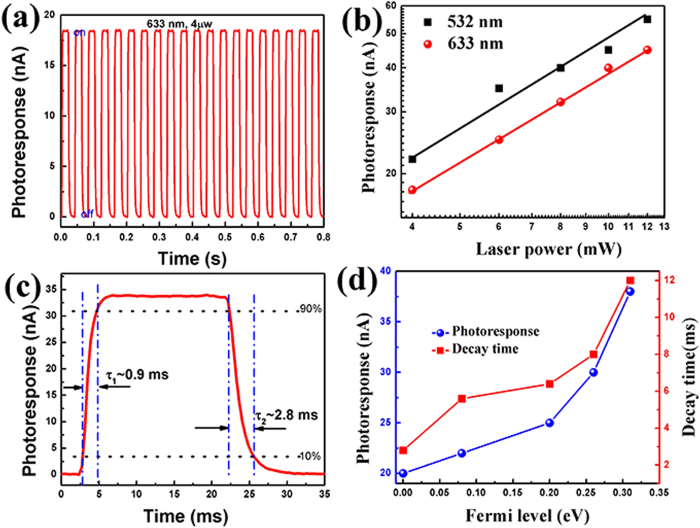
Photodetection using the p-n homo-junction graphene FET. (**a**) Time dependent photocurrent measurement on the sample irradiated for 30 s with 633 nm laser (4 μW); (**b**) Power dependence of the photocurrent with 532 nm (black curve) and 633 nm (red curve) lasers; (**c**) Photocurrent measured in one period of modulation with the 633 nm laser illumination; (**d**) Photoresponse and decay time measurements of graphene with different Fermi levels, corresponding to different irradiation times as shown in Fig. 4(b).

The potential barrier built here is the force driving the separation and transportation of photo-excited electron-hole pairs and facilitates the optoelectronic applications of graphene. Figure 5(a) shows the time-dependent switching cycles of photoresponse measurement with sample 30 s irradiated samples as a demonstration at zero source-drain bias and zero gate voltage under global illumination on the whole device. The responsivity for the devices measured under a laser illumination at 633 nm is around 5 mA/W, which is higher than that obtained in the graphene/metal Schottky junction photodetectors. The photoresponse can be expressed by a power law *I*_*PC*_ = CP^γ^ (*C* is a constant and *P* is the illumination power) as shown in Fig. 5(b). For the laser with the wavelength of 533 nm and 633 nm, γ is 0.74 and 0.78, respectively, indicating that the recombination kinetics of photocarriers involves both traps states and interactions between photogenerated carriers^33^. The decrease of the photocurrent with the incident laser power can be attributed to the reduction of the numbers of photogenerated carriers available for extraction under high photon flux due to the Auger process or the saturation of recombination/trap states that influence the lifetime of the generated carriers^34^,^35^. The external quantum efficiency of ~10% for this device is mainly limited by the insufficient absorption of incident light and the trapping and recombination of carriers as the created potential barrier is relatively small. Further increase of the barrier potential by increasing irradiation time may significantly boast the photoresponsivity and response time as shown in Fig. 5(d). On the other hand, the defect and vacancy play an essential role in the response speed of the photodetector. As shown in Fig. 5(c,d), the decay time increases with the upshift of Fermi level that represents the increase of defect density, indicating that the irradiation induced localized states leads to carrier scattering and decreases the carrier mobility. Though the response speed is slower than pure graphene photodetectors with a response time in the picosecond level, the response time and decay time are still shorter than the nanostructured disordered graphene photodetectors^5^,^33^,^36^ in which the response speed is around hundreds of seconds caused by the defect/edge scatterings and recombinations.

Another key figure of merit for a photodetector is the detectivity, which is defined as the ability to distinguish photoresponse signal from noise. In our devices, all the photoresponse measurements are conducted without source-drain bias as the photoexcited carriers can be separated by the built-in potential in the p-n junction, resulting in an extremely low dark current and invoking a high detectivity in the range of 3 × 10^10^ Jones (cm Hz^1/2^ W^−1^) for both 633 nm and 532 nm laser illuminations (see methods), which is in the same level as these for conventional semiconductor phototransistors.

## Conclusions

To summarize, we investigated the electron irradiation effect on graphene FETs and its optoelectronic application as a photodetector. The energetic electron collision induces defects on graphene sheet and modifies the substrate band bending, resulting in localized n-type doping of graphene. A potential barrier is built between the un-irradiated and irradiated regimes efficiently separating the photo-excited electron-hole pairs. On the other hand, the energetic electron/graphene collision reduces the carrier mobility and photoresponse speed of graphene based photodetectors. This work highlights the importance of the irradiation created potential barrier for graphene photodetectors and opens new venues for exploring graphene based optoelectronic devices.

## Methods

Single layer graphene flakes were mechanically exfoliated from a crystal of highly oriented pyrolytic graphite (HOPG) using adhesive 3M-tape and deposited on a silicon wafer with a 285 nm thermalized SiO_2_ layer. The silicon substrate was heavily doped p-type silicon which was employed as a back gate electrode. The location and quality of graphene was identified by optical contrast using an optical microscope and Raman spectroscopy^37^. Raman spectrum was carried out with a 532 Raman system (WITec CRM200), the laser power was kept less than 0.5 *mW* to avoid the laser induced damage of the graphene sample.

Electron beam irradiation was conducted with a JEOL field-emission SEM system under high vacuum (less than 10^−6^ Torr) under a voltage of 20 kV. The emission current was kept at 10 μA, which corresponded to a ~10 pA beam current under our experimental condition. The SEM images were taken under a low voltage (~1 kV) to reduce the irradiation effect. Soon after the location of the sample, the irradiation process was carried out with a selected area of 20 × 20 *μm*^2^ focused by the electron gun and covered part of the FET channel. The electron irradiation dosage (DOS) was controlled by the exposing time as the electron flux was kept constant during all experiments. The dose density for our device was ~n × 40 e/nm^2^ after n times of scans (every scan lasts 10 s). AFM (Dimension 3100 with nanoscope ІІІa controller, Veeco, CA, USA) was used to image the un-exposed and exposed graphene samples in taping mode in air by measuring the surface morphology and height profiles. The electrical characteristics were examined by a semiconductor analyzer (Agilent, B1500A). The photoresponsivity measurement was performed in a digital deep level transient spectroscopy (BIORAD) system with different lasers to illuminate the whole devices. The gain and detectivity are calculated as following^5^:


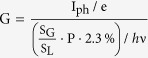
 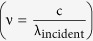 and 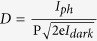, where *I*_*ph*_ and *I*_*dark*_ are the photocurrent dark current, *S*_*G*_ and *S*_*L*_ are the area of the graphene FET channel and light spot, *P* is the illumination power, υ and λ are the frequency and wavelength of the incident light, e is the electron charge, h is the Planck constant, and c is the speed of light.

## Additional Information

**How to cite this article**: Yu, X. *et al.* Photocurrent generation in lateral graphene p-n junction created by electron-beam irradiation. *Sci. Rep.* **5**, 12014; doi: 10.1038/srep12014 (2015).
